# An isocorydine derivative (d-ICD) inhibits drug resistance by downregulating IGF2BP3 expression in hepatocellular carcinoma

**DOI:** 10.18632/oncotarget.4438

**Published:** 2015-07-10

**Authors:** Meng Li, Lixing Zhang, Chao Ge, Lijuan Chen, Tao Fang, Hong Li, Hua Tian, Junxi Liu, Taoyang Chen, Guoping Jiang, Haiyang Xie, Ying Cui, Ming Yao, Jinjun Li

**Affiliations:** ^1^ School of Basic Medical Science, Shanghai Medical College, Fudan University, Shanghai, China; ^2^ State Key Laboratrory of Oncogenes and Related Genes, Shanghai Cancer Institute, Renji Hospital, Shanghai Jiaotong University School of Medicine, Shanghai, China; ^3^ Key Laboratory of Chemistry of Northwestern Plant Resources and Key Laboratory Fornatural Medicine of Gansu Province, Lanzhou Institute of Chemical Physics, Chinese Academy of Sciences, Lanzhou, China; ^4^ Qi Dong Liver Cancer Institute, Qi Dong People's Hospital, Qi Dong, Jiangsu Province, China; ^5^ Department of General Surgery, the First Affiliated Hospital, School of Medicine, Zhejiang University, Hangzhou, China; ^6^ Cancer Institute of Guangxi, Guangxi Medical University, Nanning, China

**Keywords:** isocorydine, IGF2BP3, cancer stem cell, CD133, hepatocellular carcinoma

## Abstract

In our previous studies, we reported that CD133^+^ cancer stem cells (CSCs) were chemoresistant in hepatocellular carcinoma (HCC) and that isocorydine treatment decreased the percentage of CD133^+^ CSCs. Here, we found that a derivative of isocorydine (d-ICD) inhibited HCC cell growth, particularly among the CD133^+^ subpopulation, and rendered HCC cells more sensitive to sorafenib treatment. d-ICD inhibited IGF2BP3 expression in a time-dependent manner, and IGF2BP3 expression negatively correlated with d-ICD-induced growth suppression. IGF2BP3 overexpression enriched the CD133^+^ CSC subpopulation in HCC, enhanced tumor sphere formation and suppressed the cytotoxic effects of sorafenib and doxorubicin. The expression of drug resistance-related genes, including ABCB1 and ABCG2, and the CSC marker CD133 expression was increased after IGF2BP3 overexpression. The significance of these observations was underscored by our findings that high IGF2BP3 expression predicted poor survival in a cohort of 236 patients with HCC and positively correlated with ABCG2 and CD133 expression *in vivo*. These results suggested that the d-ICD may inhibit HCC cells growth by IGF2BP3 decrease and that IGF2BP3 may serve as a therapeutic target for HCC.

## INTRODUCTION

Hepatocellular carcinoma (HCC) is one of the most common forms of cancer worldwide, and the prognosis of HCC patients is startling low unless the disease is diagnosed early [1]. However, in spite of its growing prevalence, efficient therapies are dismayingly limited. Beyond physical approaches such as radiation and surgery, few therapies are recognized, and a short list of expensive candidate drugs that display a high failure rate exists. One of the obstacles to the effective treatment of HCC is its resistance to a wide spectrum of chemotherapies.

Cancer stem cells (CSCs) contribute to drug resistance during the treatment of HCC [2]. CD133 is a HCC CSC marker. A small population of CD133^+^ cells in HCC cell lines and in primary HCC tissues mediates the high tumorigenicity and clonogenicity of HCC and upregulates the expression of multiple drug resistance-related genes [3]. Cells that are double-positive for CD133 and CD44 are more resistant to chemotherapeutic agents due to the upregulation of ATP-binding cassette (ABC) superfamily transporters, including ABCB1 and ABCG2 [4]. Additionally, a positive correlation between ABCG2 expression and the drug resistance of HCC cell lines has been confirmed. ABCG2 is preferentially expressed in highly chemoresistant HCC cancer stem cells with enriched CD133 expression [5]. Thus, targeting CD133^+^ CSCs displaying high tumorigenicity and chemoresistance is definitely necessary for the development of efficient anti-cancer strategies for HCC.

Insulin-like growth factor II mRNA-binding protein 3 (IGF2BP3, IMP-3) is considered an oncofetal protein because of its time-dependent expression in human fetal tissues but not in adult tissues [6]. The expression of IGF2BP3 is strongly associated with advanced tumor stage and is a predictor of poor prognosis among patients with HCC as well as IGF2BP1 [7, 8]. Silencing IGF2BP3, a TLR4/NANOG–dependent gene, inhibits pluripotency genes and tumorigenesis and abrogates the chemoresistance of tumor-initiating cells [9]. In addition, its expression in triple-negative breast cancer cells, which are resistant to many chemotherapeutics, increased their sensitivity to doxorubicin and mitoxantrone significantly by directly binding to ABCG2 mRNA and regulating ABCG2 expression [10]. Our previous study showed that isocorydine (ICD) treatment significantly decreased the percentage of CD133^+^ cells in HCC [11]. However, the effective concentration of ICD was too high to be tested in a clinical trial [12, 13]. To improve the anticancer activity of ICD, ten ICD derivatives were tested. Among these derivatives, 8-amino-ICD (d-ICD) exerted an inhibitory effect on murine hepatoma H22-induced tumors and was selected for further analysis [14]. Our research here showed that d-ICD treatment inhibited HCC cell growth and sensitized cancer cells to sorafenib. Further investigation indicated that d-ICD diminished the CD133^+^ CSC subpopulation in HCC and downregulated ABCB1, ABCG2 and CD133 expression via IGF2BP3 suppression.

## RESULTS

### d-ICD inhibited the growth of HCC cells, particularly in the CD133^+^ subpopulation

**Figure 1:**
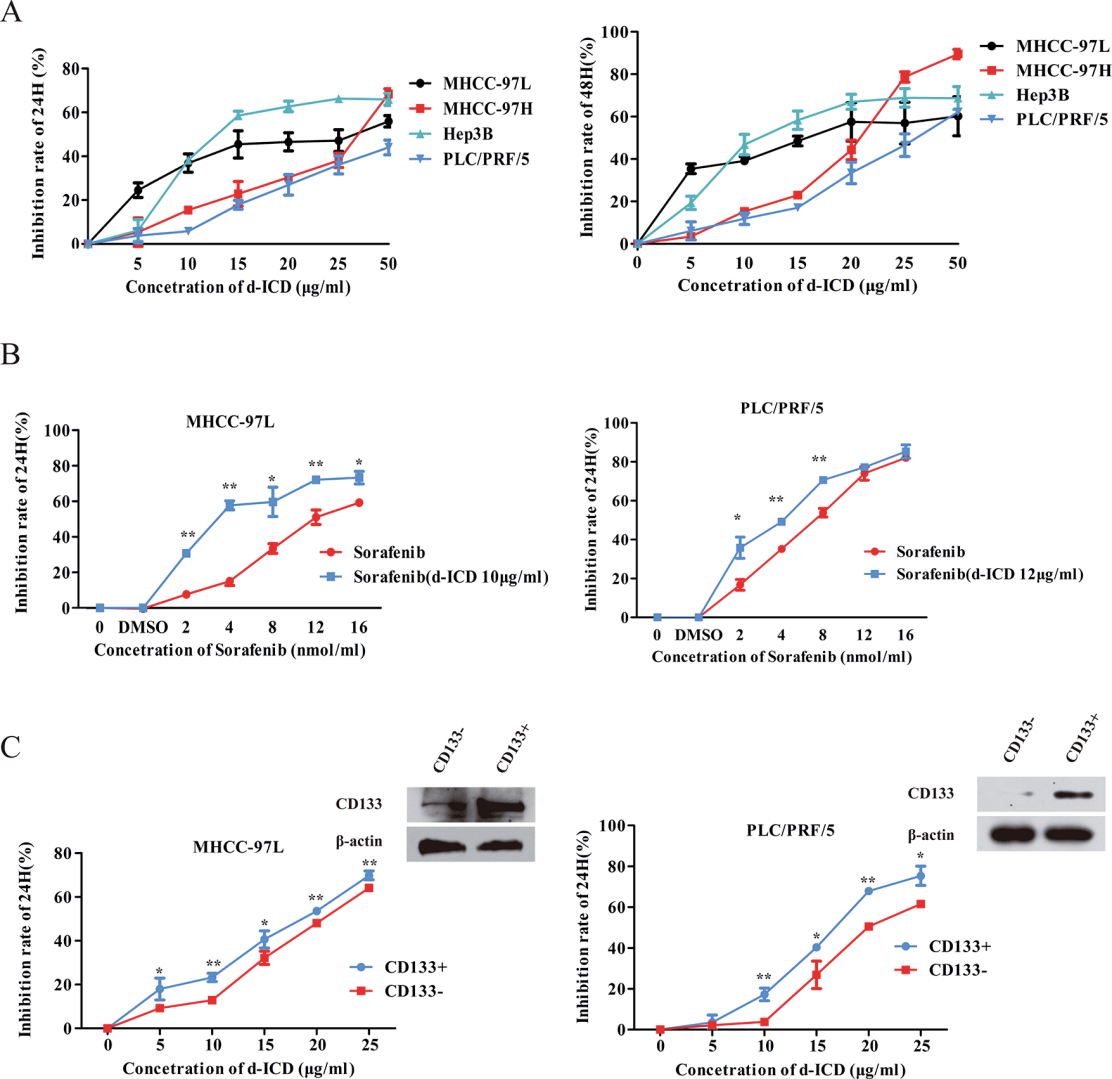
d-ICD inhibited HCC cells growth **A.** Growth inhibition induced by the treatment of HCC cells with d-ICD for 24 h and 48 h. **B.** Growth inhibition induced by the treatment of MHCC-97L and PLC/PRF/5 cells with sorafenib combining with low dose of d-ICD for 24 h. (values were represented as the mean ± SD; *p < 0.05, **p < 0.01; *t*-test, *vs* cells treated with sorafenib only) **C.** Growth inhibition induced by the treatment of CD133^+^ and CD133^−^ HCC cells with d-ICD for 24 h. (values were represented as the mean ± SD; *p < 0.05, **p < 0.01; Student *t* test, *vs* corresponding CD133^−^ cells group).

To investigate the effect of d-ICD on HCC cell growth, the HCC cell lines MHCC-97L, MHCC-97H, Hep3B and PLC/PRF/5 were incubated with various concentrations of d-ICD for 24 to 48 h. Then, cell growth was measured by the MTT assay. Our results indicated that d-ICD inhibited HCC cell growth in a dose- and time-dependent manner similar to ICD (Figure 1A). Notably, the effective dose of d-ICD dramatically decreased in these HCC cell lines compared with the effective dose of ICD, which is approximately 200 μg/ml in HCC cells [11], almost ten times the effective dose of d-ICD. Moreover, the colony formation assay confirmed that Huh7 and MHCC-97L cell growth was suppressed by d-ICD (Supplementary Figure S1).

Because sorafenib is currently a commonly used anti-HCC drug in clinics, we analyzed the effect of a low d-ICD dose, which inhibition ratio was approximately 10% in the corresponding cell line, combined with different doses of sorafenib in HCC cells to determine whether these compounds act synergistically. MTT assays performed 24 h after drug treatment showed that the MHCC-97L, PLC/PRF/5 and Huh7 cells were more sensitive to this drug combination compared with sorafenib treatment alone, indicating that this low dose of d-ICD enhances the cytotoxic effect of sorafenib on HCC cells *in vitro* (Figure 1B, Supplementary Figure S1).

We also examined the sensitivity of the CD133^+^ subpopulation to d-ICD. A magnetic-activated cell sorting experiment was performed to separate the CD133^+^ and CD133^−^ subpopulations in PLC/PRF/5, MHCC-97L and Huh7 cells. After the cells were treated for 24 h, d-ICD exerted stronger cell growth inhibition on both the PLC/PRF/5, MHCC-97L and Huh7 CD133^+^ cell populations than the corresponding CD133^−^ cell populations (Figure 1C, Supplementary Figure S1). CD133 expression in different subpopulations was confirmed by western blot analysis to establish the separation efficiency. These results demonstrated that d-ICD exerts an effect similar to that of ICD on cell growth inhibition and CD133^+^ subpopulation depletion in HCC cell lines. Moreover, we found that d-ICD sensitizes HCC cells to sorafenib treatment.

### IGF2BP3 downregulation contributed to the suppression of d-ICD-induced drug resistance

**Figure 2:**
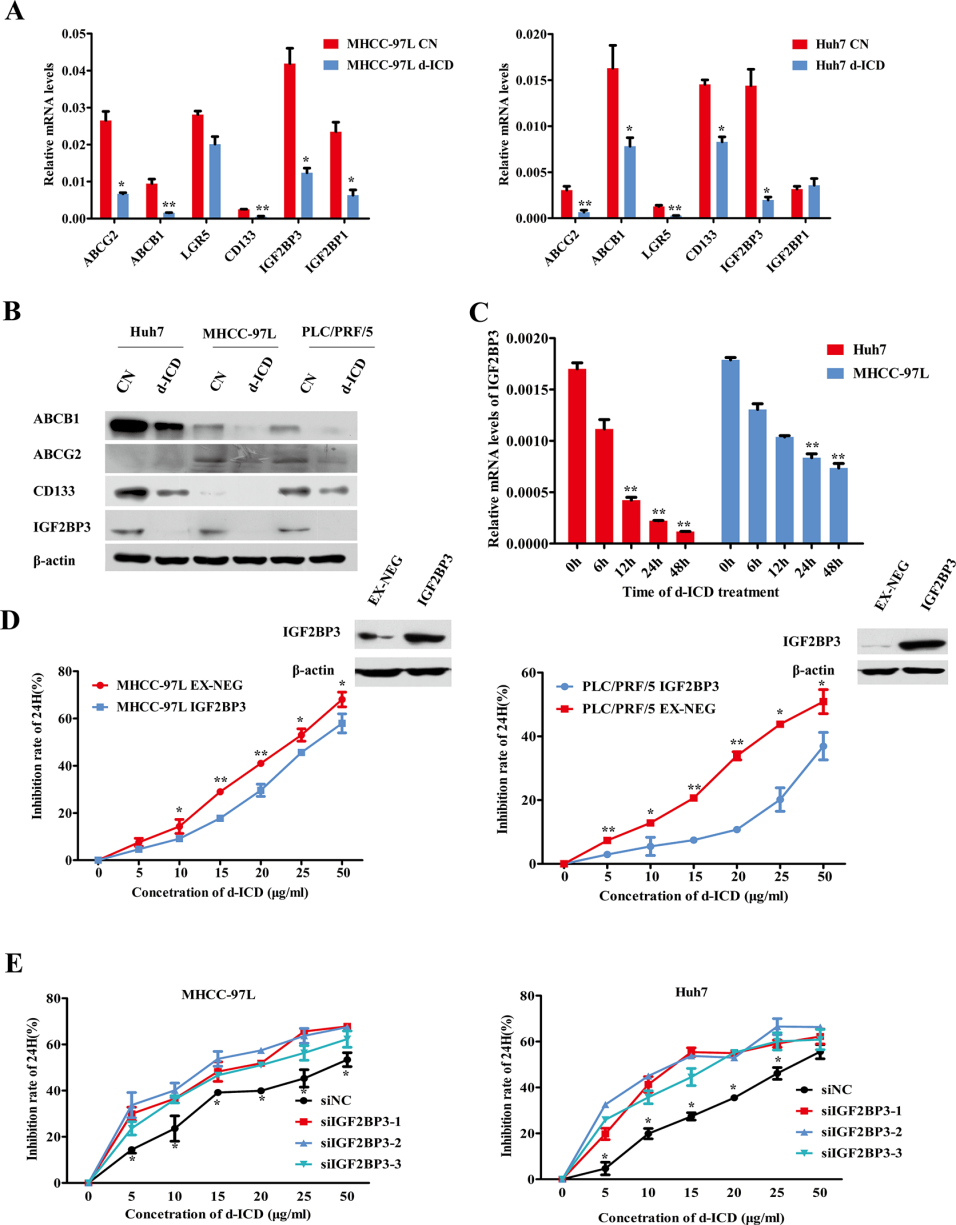
IGF2BP3 decrease facilitated drug-resistance suppression **A.** Real-time PCR analysis verified the changes of the genes expression after d-ICD treatment, which was revealed by microarray, compared with the control group. The values were shown as the mean ± SD. (*p < 0.05, **p < 0.01; Student *t* test, *vs* the control group). **B.** Western blot analysis of the genes expression with d-ICD treatment in HCC cells. **C.** IGF2BP3 mRNA expression gradually decreased after exposure to 15 ug/ml d-ICD in Huh7 and 20 ug/ml d-ICD in MHCC-97L cells in a time dependent way. (*p < 0.05, **p < 0.01; Student *t* test, *vs* the cells untreated) **D.** Growth inhibition induced by d-ICD treatment for 24 h of HCC cells with IGF2BP3 overexpression. (*p < 0.05, **p < 0.01; Student *t* test, *vs* the cells infected with the EX-NEG vector lenti-virus) **E.** IGF2BP3 targeted down-regulation promoted growth inhibition induced by the treatment of d-ICD for 24 h. (*p < 0.05, **p < 0.01; Student *t* test, *vs* the cells transfected with stable negative control siRNA oligonucleotides, siNC for short).

To further evaluate the molecular mechanism by which d-ICD inhibited cell growth in HCC, we applied cDNA microarrays to detect the differentially expressed genes in Huh7 and SMMC-7721 cells between the control and d-ICD treatment groups. The resulting genes were verified *via* RT-PCR assays, and we confirmed the deregulation of several genes in Huh7 cells due to d-ICD treatment (Supplementary Table S1). Among these candidate genes, we found that the drug resistance-related genes ABCB1 and ABCG2, as well as the stemness-related genes CD133, Lgr5, IGF2BP3 and IGF2BP1, were downregulated at the mRNA level, and CD133, IGF2BP3, ABCB1 and ABCG2 protein expression were also downregulated following d-ICD treatment (Figure 2A and 2B). Among these genes, IGF2BP3 mRNA expression gradually decreased after exposure to d-ICD in Huh7, MHCC-97L and PLC/PRF/5 cells in a time-dependent manner (Figure 2C, Supplementary Figure S1).

To study the role of IGF2BP3 in d-ICD treatment, we first analyzed intrinsic IGF2BP3 expression in several HCC cell lines to select appropriated cell lines for the following analysis, and CD133 expression in different cell lines was also taken in consideration [4] (Supplementary Figure S2). Then, IGF2BP3 was ectopically overexpressed in PLC/PRF/5, Huh7 and MHCC-97L cells and confirmed by western blot analysis (Figure 2D). The MTT assay results revealed that the MHCC-97L-IGF2BP3-EX-NEG (MHCC-97L-IGF2BP3) and PLC/PRF/5-IGF2BP3-EX-NEG (PLC/PRF/5-IGF2BP3) cells were less sensitive to various doses of d-ICD than those of the control cells infected with the EX-NEG vector lentivirus (Figure 2D). Conversely, as shown in Figure 2E, transfection with small interfering RNA (siRNA) oligonucleotides specifically targeting IGF2BP3 clearly enhanced the cytotoxic effects of d-ICD on Huh7 and MHCC-97L cells. The knockdown efficiency was confirmed by real-time RT-PCR (Supplementary Figure S2). IGF2BP3 protein could bind to IGF2 mRNA and promote IGF2 protein expression in human rhabdomyosarcoma cells [15]. We also analyzed IGF2 expression after IGF2BP3 was knocked down or overexpressed. Real-time PCR assay displayed that IGF2 mRNA expression was positively correlated with IGF2BP3 expression in HCC (Supplementary Figure S2). These results indicated that IGF2BP3 may be a target gene of d-ICD in HCC cells that plays a crucial role in d-ICD-induced growth inhibition.

### IGF2BP3 enriched the CD133^+^ CSC population and promoted HCC cell chemoresistance

**Figure 3:**
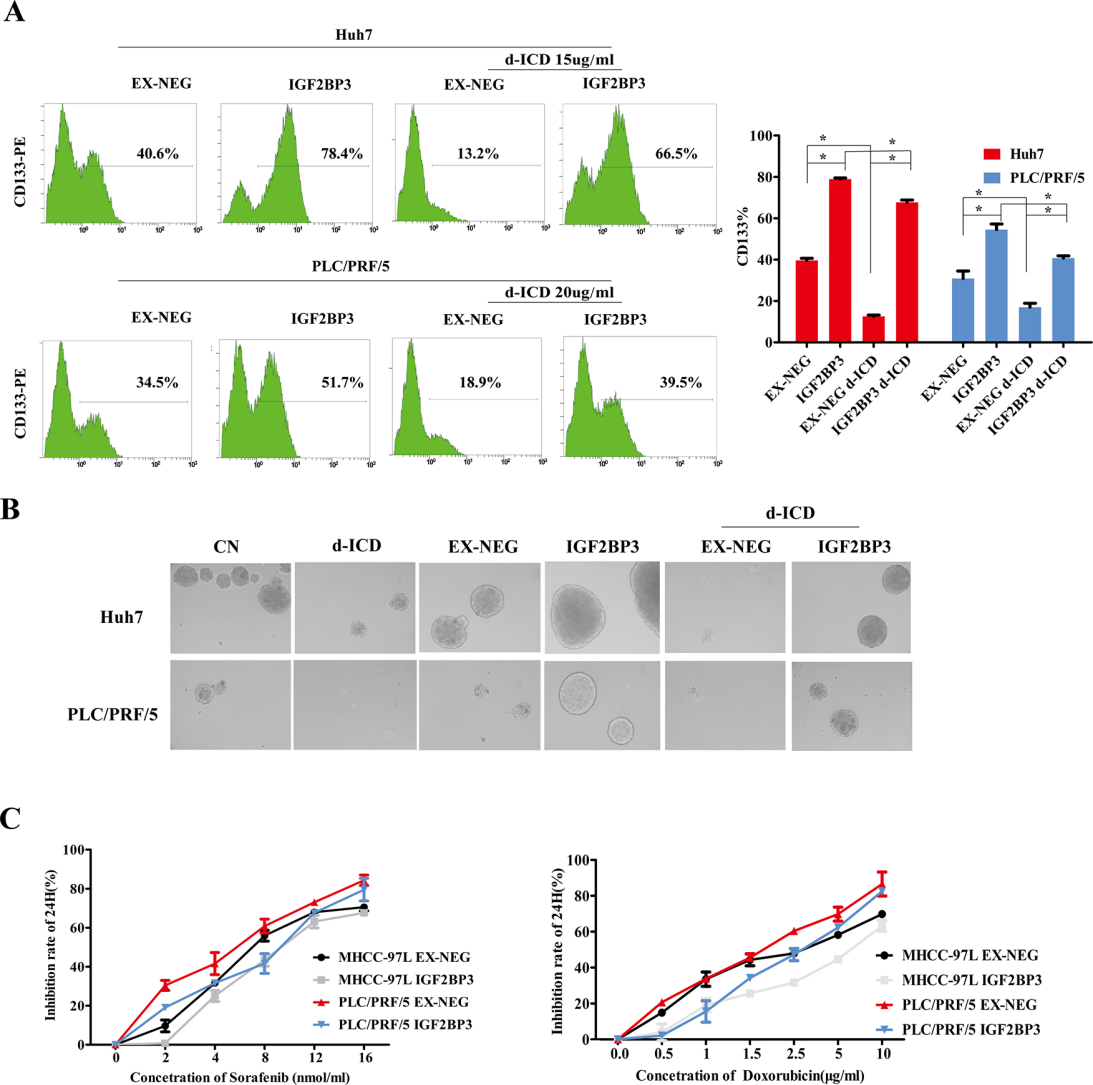
IGF2BP3 enriched CSCs population and promoted chemoresistence **A.** The percentage of CD133^+^ cells was determined by flow cytometry. Statistically significant differences were determined using the Student *t* test (*p < 0.05, **p < 0.01; *vs* the group indicated). **B.** The tumor-sphere formation ability of PLC/PFR/5 and Huh7 cells with IGF2BP3 overexpression. **C.** Growth inhibition induced by the treatment of Sorafenib and Doxorubicin for 24 h in HCC cells with IGF2BP3 overexpression.

In our previous studies, we demonstrated that CD133 is a marker of HCC CSCs. As shown in Figure 3A, FACS analysis indicated that the percentage of the CD133^+^ subpopulation in Huh7 cells decreased from 54.6% ± 2.139% to 27.3% ± 2.145% following treatment with 15 μg/ml d-ICD for 24 h. Similar results were obtained in PLC/PRF/5 cells after these cells were exposed to 20 μg/ml d-ICD for 24 h. IGF2BP3 overexpression increased the CD133^+^ cell population in both cell lines, and the d-ICD-induced depletion of this cell population was attenuated by IGF2BP3 overexpression.

Next, self-renew is an important feature of CSCs. We investigated the self-renewal capability of the IGF2BP3-overexpressing HCC cells using a sphere formation assay. The tumor spheres obtained from d-ICD-treated Huh7 cells continually cultured in stem cell conditioned chemically defined medium (CDM) were fewer in number and smaller in size than those from the control cells. After IGF2BP3 overexpression, the tumor sphere formation ability of these cells was significantly enhanced, and d-ICD treatment did not completely abolish this effect, indicating that IGF2BP3 overexpression enhances the self-renewal capability of HCC cells and partially reverses the inhibitory effects of d-ICD. We observed similar results using the PLC/PRF/5 cell line (Figure 3B).

To analyze the chemotherapy drug sensitivity of IGF2BP3-overexpressing HCC cells, we designed MTT assays to test the efficacy of sorafenib and doxorubicin. Compared to the control conditions, the overexpression of IGF2BP3 rendered Huh7 and PLC/PRF/5 cells less sensitive to these two commonly used drugs. These results suggested that IGF2BP3 positively regulates HCC cell stemness and promotes HCC cell chemoresistance.

### IGF2BP3 upregulated the expression of ABC family genes and enhanced doxorubicin effluxion

**Figure 4:**
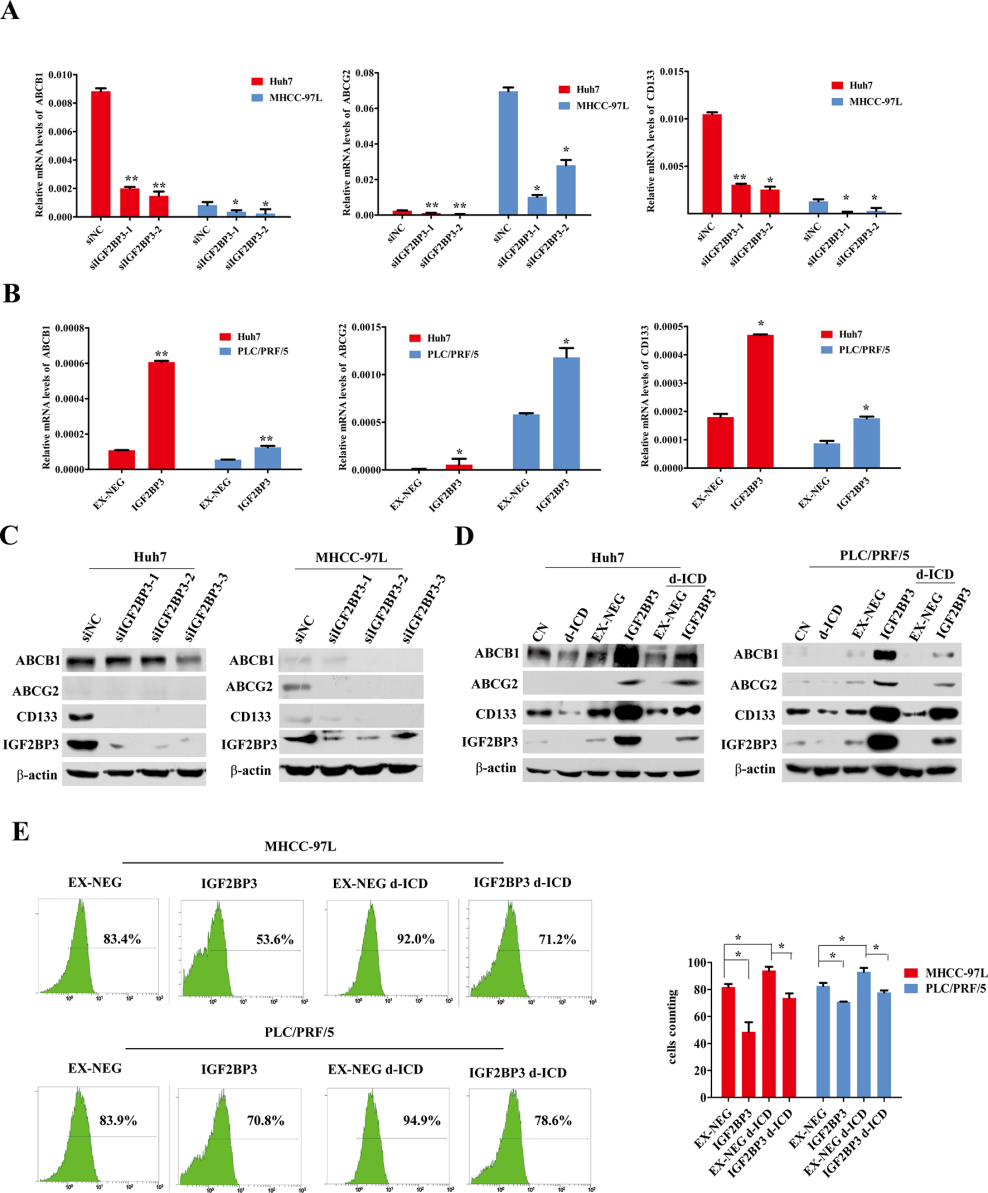
IGF2BP3 upregulated ABC familiy and CD133 genes expression **A.** Real-time PCR analysis revealed that down-regulation of IGF2BP3 leading to ABCB1, ABCG2 and CD133 mRNA expression depression in MHCC-97L and Huh7 cells. The values were displayed as mean ± SD. (*p < 0.05, **p < 0.01, Student *t* test, *vs* the cells transfected with non-coding siRNA oligonucleotides) **B.** Real-time PCR analysis showed that IGF2BP3 overexpression upregulated ABCB1, ABCG2 and CD133 mRNA expression in Huh7, MHCC-97L and PLC/PRF/5 cells. The values were presented as mean ± SD. (*p < 0.05, **p < 0.01, Student *t* test, *vs* the cells infected with the EX-NEG vector lenti-virus) **C. and D.** IGF2BP3 knockdown and overexpression regulated ABCB1, ABCG2 and CD133 protein expression in Huh7, MHCC-97L and PLC/PRF/5 cells. **E.** FACS analysis demonstrated that the effluxion of Doxorubicin in MHCC-97L and PLC/PRF/5 cells were enhanced by IGF2BP3 overexpression. d-ICD treatment promoted the retention of doxorubicin, and IGF2BP3 overexpression partially reversed d-ICD effect. (*p < 0.05, **p < 0.01, Student *t* test, *vs* the group indicated).

The downregulation of IGF2BP3 led to the suppression of ABCB1, ABCG2 and CD133 mRNA expression in MHCC-97L and Huh7 cells (Figure 4A). IGF2BP3 overexpression deregulated ABCB1, ABCG2 and CD133 mRNA expression in Huh7, MHCC-97L and PLC/PRF/5 cells (Figure 4B), and we observed a moderate upregulation of the expression of the stemness-related genes Nanog and Oct-4 in HCC cells (Supplementary Figure S3). Western blot analysis indicated a similar expression pattern at the protein level (Figure 4C and 4D). Similar to the results shown in Figure 2B, western blot analysis indicated that d-ICD treatment decreased ABCB1, ABCG2 and CD133 protein expression but that IGF2BP3 upregulation nearly completely abolished this effect of d-ICD on HCC cells (Figure 4D). These results demonstrated that ABCB1, ABCG2 and CD133 may be downstream target genes of IGF2BP3 in HCC.

The anticancer drug doxorubicin is commonly used in HCC chemotherapy and is a naturally fluorescent molecule that can be transported by ABCB1 and ABCG2. Our previous study demonstrated that the intracellular concentration of doxorubicin can be monitored *via* flow cytometry [16]. As shown in Figure 4E, IGF2BP3 overexpression enhanced the effluxion of doxorubicin in MHCC-97L and PLC/PRF/5 cells. d-ICD treatment promoted the retention of doxorubicin, and IGF2BP3 overexpression partially reversed this effect of d-ICD. These results clearly indicated that the active drug efflux ability of the endogenous ABCB1 and ABCG2 proteins correlated with IGF2BP3 expression in HCC and was suppressed by d-ICD treatment.

### The IGF2BP3 expression level correlated with adverse clinicopathological characteristics in HCC patients

**Figure 5:**
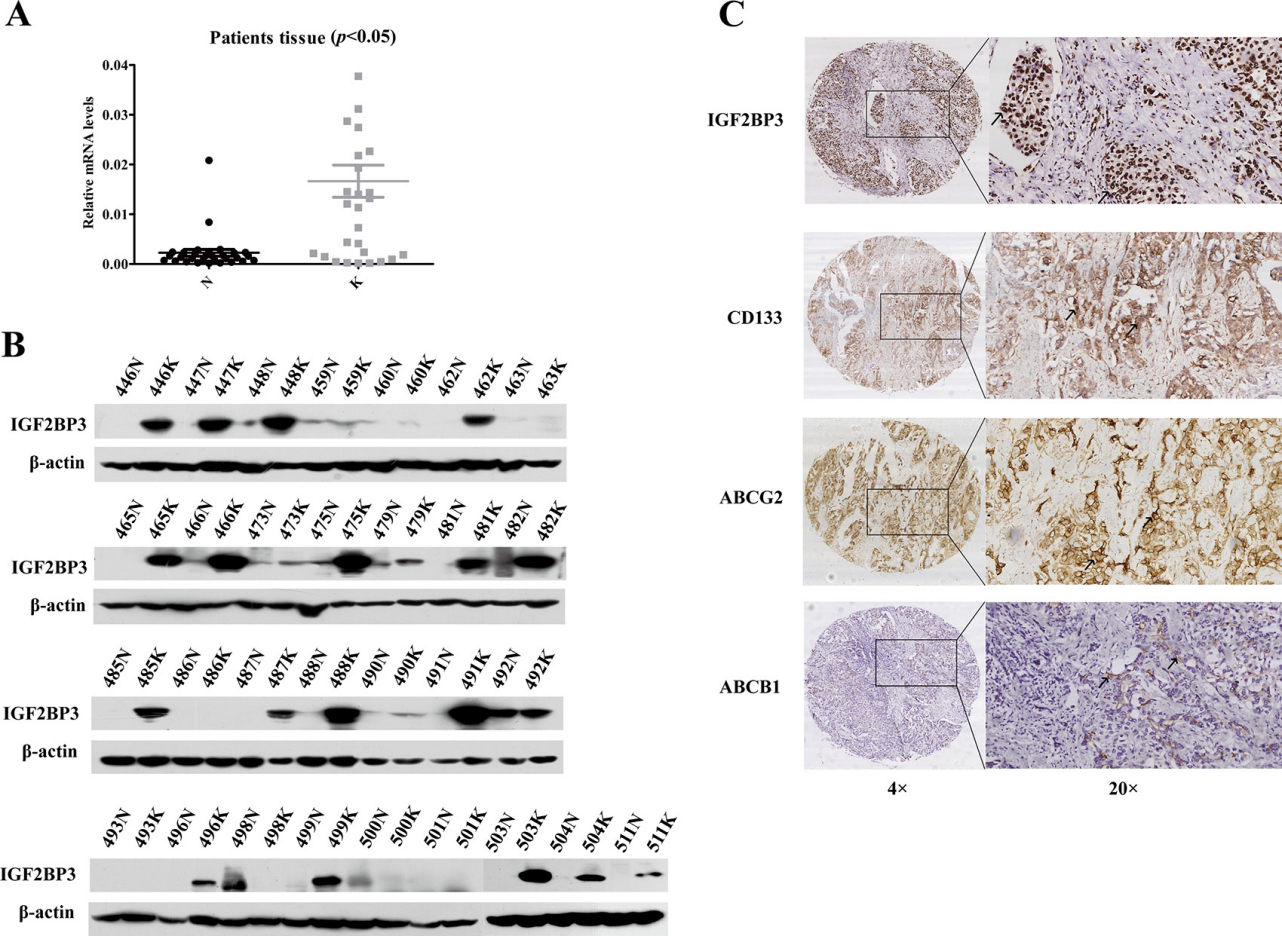
IGF2BP3 expression correlated with adverse prognosis in HCC patients The expression of IGF2BP3 at mRNA **A.** and protein **B.** level in 30 pairs of patients samples. **C.** The IHC results of IGF2BP3, ABCG2 and CD133 expression in HCC samples. Representative images are shown. The arrows point to the representative positive staining cells in the images. Original magnification of pictures, 200×.

In 30 paired tumor and adjacent noncancerous liver tissues from HCC patients, we analyzed the expression levels of IGF2BP3 *via* real-time RT-PCR and western blot. We found that the majority of tumor tissue samples expressed a higher level of IGF2BP3 than the corresponding adjacent non-cancerous tissue samples at both the mRNA (Figure 5A) and protein levels (Figure 5B) (Supplementary Figure S3), suggesting that a high level of IGF2BP3 expression is a predictor of poor prognosis in HCC patients.

Then, immunohistochemical (IHC) assays indicated that the frequency of positive staining for IGF2BP3 was 40.67% (96/236) in a cohort of 236 patients with HCC. Further analysis showed that the IGF2BP3 levels were significantly higher in the HCC patients with higher histological grades or with intrahepatic metastasis (*p* < 0.05) (Table 1). Furthermore, we analyzed CD133, ABCG2 and ABCB1 expression *via* IHC (Figure 5C). The frequencies of positive staining for CD133, ABCG2 and ABCB1 proteins were 70.33% (166/236), 50% (118/236), and 63.14% (169/236), respectively, and IGF2BP3 expression positively correlated with both CD133 and ABCG2 expression (*p* < 0.05), which suggested that IGF2BP3 regulates CD133 and ABCG2 expression *in vivo* (Table 2).

**Table 1:** Relationship between IGF2BP3 expression and clinicopathological features in HCC tissues

Clinicopathological Features	Number of cases	IGF2BP3 immunostaining^#^		
		Score 0 *N* (%)	Score 1 *N* (%)	*P* Value
Age (years)				
<60	159	97 (69.29)	62 (65.26)	0.518
≥60	76	43 (30.71)	33 (34.74)	
Gender				
Male	190	109 (77.86)	81 (84.38)	0.214
Female	46	31 (22.14)	15 (15.62)	
Tumor size				
≤5 cm	113	66 (47.83)	47 (51.65)	0.571
>5 cm	116	72 (52.17)	44 (48.35)	
AFP (ng/ml)				
≤20	79	51 (37.5)	28 (29.17)	0.187
>20	153	85 (62.5)	68 (70.83)	
HBV infection				
Negative	42	28 (20.74)	14 (14.89)	0.261
Positive	187	107 (79.26)	80 (85.11)	
Cirrhosis				
Absent	38	25 (17.86)	13 (13.54)	0.376
Present	198	115 (82.14)	83 (86.46)	
Edmondson's grade				
I, II	119	78 (55.71)	41 (42.71)	0.050*
III, IV	117	62 (44.29)	55 (57.29)	
Intrahepatic metastasis				
Absent	161	105 (75.00)	56 (58.33)	0.007*
Present	75	35 (25.00)	40 (41.67)	

AFP, alpha-fetoprotein. N, Number of cases. *p* value represents the probability from a Chi-square test for different immunohistochemical scores of IGF2BP3 in HCC tissues.

* *p* < 0.05.

# Score 0 and Score 1 respectively indicate weak and strong immunostaining.

**Table 2:** Correlation between IGF2BP3, CD133, ABCG2 and ABCB1 protein

	IGF2BP3 immunostaining			
	Score 0	Score 1	*r* value	*p* value
**IGF2BP3 immunostaining**				
**Score 0**	140	0		
**Score 1**	0	96		
**CD133 immunostaining**				
**Score 0**	51	19	0.179	0.006*
**Score 1**	89	77		
**ABCG2 immunostaining**				
**Score 0**	102	16	0.552	0.000*
**Score 1**	38	80		
**ABCB1 immunostaining**				
**Score 0**	49	38	–0.047	0.473
**Score 1**	91	58		

*P* value represents the probability from a Chi-square test for different immunohistochemical scores of IGF2BP3 in HCC tissues.

* *p* < 0.05.

## DISCUSSION

Among the five primary components of *Dicranostigma leptopodum* (Maxim) Fedde (DLF) extracts, we found that ICD inhibits the proliferation of HCC cells [17]. The mechanism underlying the anti-tumor properties of ICD included its induction of a significant decrease in the CD133^+^ cell subpopulation, an effect that traditional chemotherapy drugs were unable to accomplish [11].

Here, we showed that d-ICD exerted a relatively stronger inhibitory effect on the CD133^+^ cell population than the corresponding CD133^−^ cell population at a much lower effective dose than its parent compound ICD. Furthermore, d-ICD treatment remarkably inhibited the tumorigenicity of Huh7 cells, which contain a relatively high percentage of CD133^+^ cells (data to be published elsewhere). Based on cDNA microarray analysis, we discovered a key d-ICD target, IGF2BP3. A previous study showed that IGF2BP3 expression significantly correlated with CD44s expression, and the expression levels of both IGF2BP3 and CD44s correlated with advanced tumor stage and grade in HCC [18]. Several studies addressing IGF2BP3 expression in colorectal cancer have detected significantly elevated IGF2BP3 expression in the majority of aggressive colorectal carcinomas (CRCs), suggesting that IGF2BP3 expression correlates with an unfavorable prognosis. Similarly, the present study revealed that high levels of IGF2BP3 expression correlated with both Edmondson's grade and intrahepatic metastasis in HCC, corresponding to an adverse prognosis.

Previous studies have demonstrated that CSCs have a particularly regenerative capacity; thus, this property must be inhibited to achieve the stable remission or even a cure for cancer [19]. Self-renewal may be the most important and useful property of CSCs. Our results showed that d-ICD treatment effectively inhibits CD133 expression via IGF2BP3 downregulation. Furthermore, d-ICD treatment may decrease the percentage of CD133^+^ CSC cells and suppress their self-renew capability, which is believed to contribute to drug resistance. CSCs from various tumor types characteristically express drug resistance-related proteins, particularly ABC family proteins [20, 21]. ABCG2 is closely associated with chemotherapy drug resistance and is highly expressed in enriched CD90^+^/CD133^+^ liver CSCs. Our previous studies showed that high ABCG2 expression in liver CSCs may be the cause of high drug resistance in liver cancer [4, 5]. The development of multidrug resistance (MDR) in cancers is also associated with the overexpression of ABCB1 [22]. ABCB1, which exports many conventional chemicals, particularly doxorubicin, out of cells and renders chemotherapy inefficient [23], may also display a close relationship with the resistance to clinically relevant chemotherapies. ABCB1 overexpression occurs in various types of tumors [24], and high ABCB1 expression is associated with poor prognosis in patients with acute lymphoblastic leukemia [24, 25]. We found that IGF2BP3 overexpression led to ABCB1 and ABCG2 upregulation, which may result in resistance to many conventional chemicals. Additionally, IGF2BP3 overexpression led to the enrichment of the CD133^+^ subpopulation, which may contribute to ABCG2 and ABCB1 upregulation. We had also found a positive correlation between IGF2BP3, ABCG2 and CD133 expression *in vivo*. However there was no significant correlation between IGF2BP3 and ABCB1 expression observed, indicating that the regulation effect of IGF2BP3 on ABCB1 expression in HCC cells may be complementary *in vivo*. Therefore, targeting IGF2BP3 in HCC cells, but not in normal cells, which can express barely detectable levels of IGFP2BP3, may be a more effective treatment.

IGF2BP3 was previously reported to be preferentially expressed in triple-negative breast cancers, which exhibit multi-drug resistance and increased aggressiveness. IGF2BP3 binds to BCRP mRNA and regulates BCRP expression; this finding provides insight into the mechanism by which IGF2BP3 contributes to the aggressiveness of cancers [25]. Functional studies addressing the regulatory role of IGF2BP3 revealed two validated target mRNAs and several putative candidates, including IGF2 and ABCG2. Two laboratories reported that IGF2BP3 regulates the translation of IGF2 mRNA [26, 27]. However, in this study, we had not verified the detailed mechanism underlying IGF2BP3 function in HCC. Whether IGF2BP3 also directly binds to ABCG2, CD133 or ABCB1 mRNA to regulate their expression at the post-transcription level remains to be investigated in our future studies. Another possible way that IGF2BP3 regulate these genes expression may rely on its regulation of IGF2 [15]. We analysis the expression of IGF2 mRNA after IGF2BP3 overexpression or knocked down. The results showed that IGF2BP3 could also regulate IGF2 expression in HCC cells. Bendall et al reported that blocking IGF2 reduced survival and clonogenicity of human embryonic stem (ES) cells and IGF2 alone was sufficient in maintaining human ES cell cultures [28]. IGF2BP3 may increase the cancer stem cell percentage through IGF2 in HCC, which probably lead to the stemness-related genes like CD133 and ABCB1 expression up-regulation.

Taken together, IGF2BP3 is involved in the selective effects of d-ICD on CD133^+^ HCC CSCs and represents a potential target for future HCC therapy. Our findings suggest that a combination of sorafenib and d-ICD may be a promising approach to overcoming the therapeutic resistance of human HCC.

## MATERIALS AND METHODS

### Cell lines and cell culture

The human HCC cell lines SMMC-7721 and Huh7 were provided by the Cell Bank of the Institute of Biochemistry and Cell Biology (Shanghai, China). MHCC-97L, MHCC-97H and MHCC-LM3 cells were obtained from the Liver Cancer Institute of Zhongshan Hospital at Fudan University (Shanghai, China). PLC/PRF/5 cells were purchased from the American Type Culture Collection (ATCC) (Manassas, USA). All cell lines were cultured in Dulbecco's modified Eagle's medium (Sigma-Aldrich, St Louis, MO) containing 10% fetal bovine serum (FBS) (HyClone, Logan, UT) that was heat-inactivated at 56°C for 30 min and then supplemented with 100 IU/ml penicillin G and 100 μg/ml streptomycin (Sigma). All cell lines were incubated at 37°C in a humidified atmosphere of 5% CO_2_.

### Drug stocks

Isocorydine derivates (d-ICD), prepared through chemical structure modifications of isocorydine (ICD), which was synthesized from ICD using a previously described method [29] was dissovled in PBS and diluted to 100 mg/ml in PBS and stored at 4°C in a dark container. Sorafenib tosylate (CAS No. 475207-59-1), a multikinase inhibitor, was diluted to 100 nmol/μl in DMSO and stored at 4°C.

### Fluorescence-activated cell sorting (FACS) or magnetic-activated cell sorting

The PLC/PFR/5 and Huh7 cells were incubated with PE-conjugated CD133/1 (AC133) antibody (Miltenyi Biotec, Germany), and the percentage of CD133^+^ cells was detected using an Epics Altra flow cytometer (Beckman Coulter, USA). For MHCC-97L and PLC/PFR/5 cells, the CD133^+^ and CD133^−^ cells were magnetically isolated using corresponding antibodies and an EasySep PE Selection Kit (Stem Cell Technologies) according to the manufacturer's instructions. The purity of the sorted cells was evaluated by western blot.

### Western blot analysis

Cells were lysed and subjected to SDS-PAGE. Then, the proteins were transferred to nitrocellulose or polyvinylidene difluoride (PVDF) membranes. The proteins on the membranes were incubated with specific primary antibodies and HRP-conjugated secondary antibodies (Supplementary Table S2), followed by chemiluminescence detection using a Super Signal West Femto Chemiluminescent Substrate Kit (Thermo Scientific, Cat No. 34095).

### MTT assay

First, 4000 cells/well were incubated in triplicate in 96-well plates for 24 h and then exposed to various d-ICD concentrations for 24 h or 48 h. The MTT stock solution, which was diluted to 0.5 μg/ml, was added to each well and incubated for 4 h. Finally, the crystals were dissolved in DMSO, and absorbance was measured using an ELISA plate reader. The growth inhibition rate was calculated as follows: Growth inhibition rate = 1 – (A570 – A630) of the experimental cells/(A570 – A630) of the control cells.

### RNA interference-mediated gene silencing

siRNA oligos for IGF2BP3 and a generic negative control sequence were synthesized by GenePharma (Shanghai, China). The sequences were as follows: IGF2BP3-homo-979 sense: 5′-GCUGCUGAGAAGUCGAUUATT-3′; antisense: 5′-UAAUCGACUUCUCAGCAGCT T-3′; IGF 2BP3-homo-1028 sense: 5′-CGGCUUGUAAGUCUAUU CUTT-3′; antisense: 5′-AGAAUA GACUUACAAGCCG TT-3′; and IGF2BP3-homo-1567 sense: 5′-GCUGGAGCUU CAAUUAAGATT-3′; antisense: 5′-UCUUAAUUGAAGCU CCAGCTT-3′.

### Quantitative real-time RT-PCR

RNA was extracted using TRIzol reagent (Invitrogen) according to the manufacturer's protocol and then reverse-transcribed into cDNA using a PrimeScript™ RT Reagent Kit (TaKaRa). The primers used for quantitative RT-PCR are provided in Supplementary Table S3.

### Plasmid constructs, lentivirus production, and cell transfection

The full-length human IGF2BP3 plasmid was provided by GeneCopoeia Company (US). Virus packaging and cell transfection were performed as previously described [30].

### Sphere formation assay

HCC cells were plated in generic 6-well plates (NUNC) as a monolayer culture in serum-free CDM. For d-ICD treatment, different amounts of d-ICD were added to the CDM to reach a final concentration of 15 μg/ml for Huh7 cells or 20 μg/ml for PLC/PRF/5 cells. The CDM consisted of a 1:1 mixture of neurobasal medium and DMEM/F12 medium supplemented with 0.5 × N2, 0.5 × B27 supplement, 0.1% bovine serum albumin, 2 mmol/L glutamine, and 0.1 mmol/L 2-mercaptoethanol; growth factors including 10 ng/ml basic fibroblast growth factor (Millipore), 10 ng/ml EGF (Millipore), 20 ng/mL hepatocyte growth factor (Millipore), 20 ng/ml TGFα (Millipore), and 10 × 7 mol/L dexamethasone (Sigma Aldrich) were added. The reagents that were not otherwise indicated were purchased from Invitrogen Corporation (US).

### Immunohistochemistry

All of the 236 HCC patients gave consent for the use of their tumor tissues in the study. The University Ethical Committee approved the collection of fresh tumor tissue samples for clinical analysis. Immunohistochemistry, positive staining result evaluation, and statistical data analysis were performed as our previous description [30]. The results were visualized and imaged using a slide scanner leica mod SCN400 imaging system (Leica, Germany).

### Statistical analysis

All data are presented as the mean ± standard deviation (SD). *P* < 0.05 was considered statistically significant. Statistical analyses (comparisons between two groups) were performed using Student's *t* test.

## SUPPLEMENTARY FIGURES AND TABLES


