# Supplementary material for: An isocorydine derivative (d-ICD) inhibits drug resistance by downregulating IGF2BP3 expression in hepatocellular carcinoma
Source: Oncotarget. 2015 Jul 10;6(28):25149–60. doi: 10.18632/oncotarget.4438 (PMC4694821; doi:10.18632/oncotarget.4438)
Supplement: Supplementary file 1 [file oncotarget-06-25149-s001.pdf]

SUPPLEMENTARY FIGURES AND TABLES

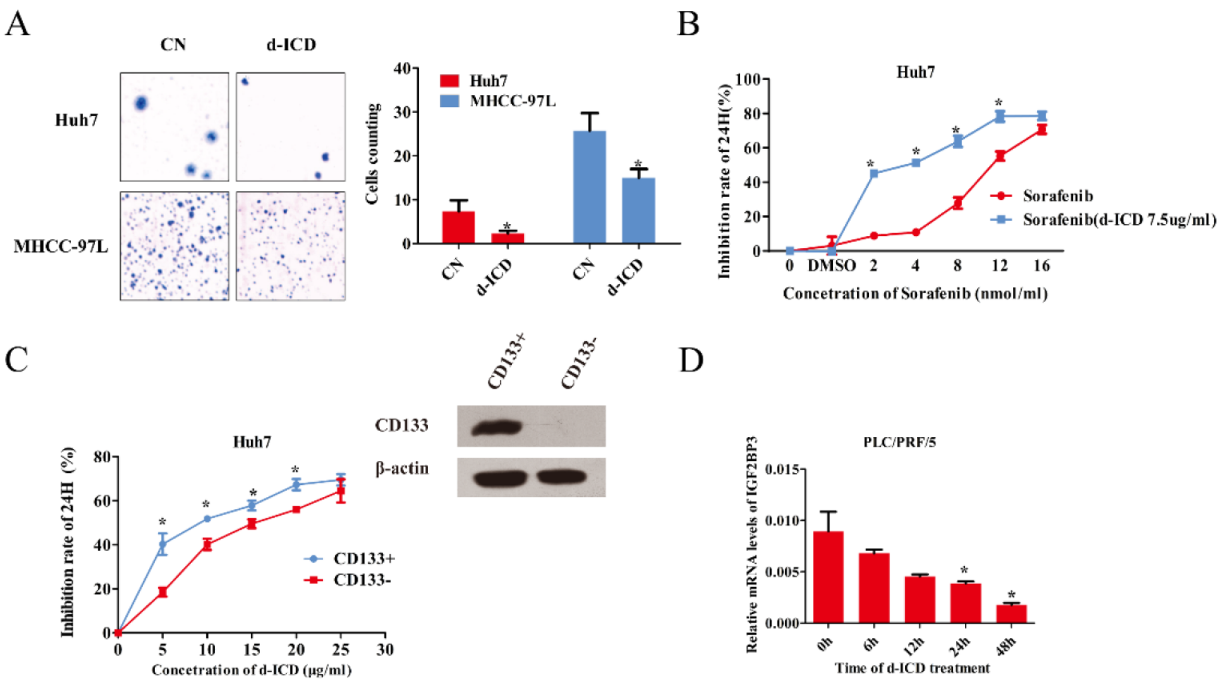

Supplementary Figure S1: A. Growth inhibition resulting from the treatment of HCC cells with d-ICD for 24 h, ****p* **< 0.05 B. Growth inhibition resulting from the treatment of Huh7 cells with Sorafenib combined with low dose of d-ICD for 24 h, ****p*** **< 0.05. C.** Growth inhibition induced by the treatment of $CD133^+$ and CD133- HCC cells with d-ICD for 24 h. (values were represented as the mean ± SD; *$p < 0.05$; Student *t* test, *vs* corresponding $CD133^-$ cells group). **D.** IGF2BP3 mRNA expression gradually decreased after exposure to 20 ug/ml d-ICD in PLC/PRF/5 cells in a time dependent way. (*$p < 0.05$; Student *t* test, *vs* the cells untreated).

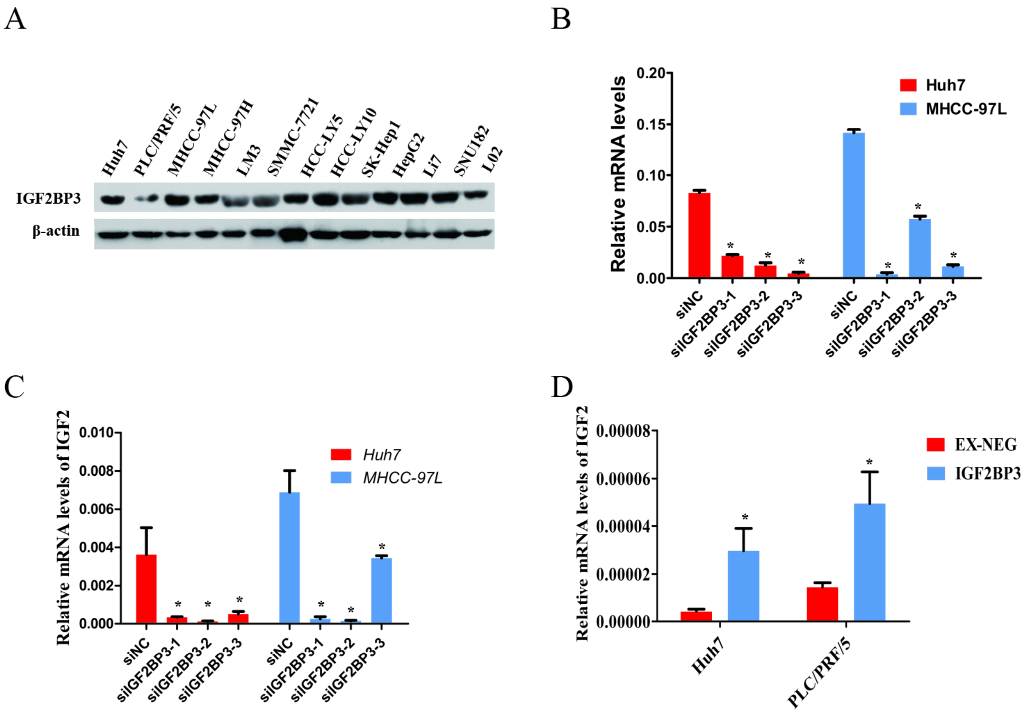

Supplementary Figure S2: A. The expression of IGF2BP3 in different cells was tested by Western Blotting. B. Real-time PCR analysis revealed down-regulation of IGF2BP3 by RNAi was successfully completed, *$p < 0.05$. **C.** Real-time PCR analysis of IGF2 expression in IGF2BP3 knock-down HCC cells. *$p < 0.05$, *t*-test. **D.** Real-time PCR analysis of IGF2 expression in IGF2BP3 overexpressed HCC cells. *$p < 0.05$, *t*-test.

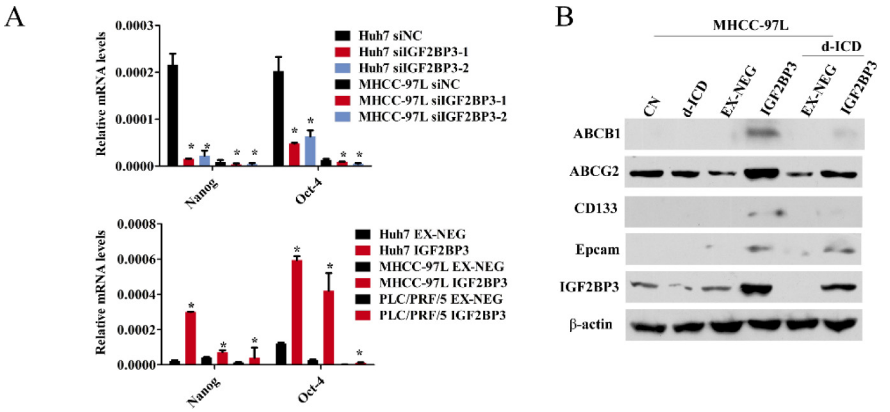

Supplementary Figure S3: A. Real-time PCR analysis showed that IGF2BP3 expression corelated with stemness-related genes Nanog and Oct-4 expression in HCC, *$p < 0.05$. B. Western bloting results displayed that d-ICD treatment decreased ABCB1, ABCG2 and CD133 protein expression, and the up-regulation of IGF2BP3 nearly abolished the depression effect of d-ICD in HCC cells.

Supplementary Table S1: cDNA microarray for the differentially expressed genes in HCC cell lines between the control and d-ICD treatment group

Gene Symbol	Gene Tile	UniGene ID	Relative Fold Change (d-ICD/CN)
Upregulated genes			
**IGF2BP1**	**insulin-like growth factor 2 mRNA binding protein 1**	**Hs.144936**	**2.0456**
ATF3	activating transcription factor 3	Hs.460	14.49
GDF15	growth differentiation factor 15	Hs.616962	7.0462
EIF1	eukaryotic translation initiation factor 1	Hs.150580	2.2575
KLF6	Kruppel-like factor 6	Hs.4055	2.346
SHMT2	serine hydroxymethyltransferase 2 (mitochondrial)	Hs.75069	2.1043
SLC16A5	solute carrier family 16, member 5(monocarboxylic acid transporter 6)	Hs.592095	2.0538
TNFRSF10B	tumor necrosis factor receptor superfamily, member 10	Hs.521456	2.0735
TRIB3	tribbles homolog 3 (Drosophila)	Hs.516826	2.8754
TSC22D3	TSC22 domain family, member 3	Hs.522074	3.2892
Downregulated genes			
**ABCB1**	**ATP-binding cassette, sub-family B (MDR/TAP), member 1**	**Hs.737655**	**0.3998**
**ABCG2**	**ATP-binding cassette, sub-family G (WHITE), member 2**	**Hs.480218**	**0.8166**
**LGR5**	**leucine-rich repeat containing G protein-coupled receptor 5**	**Hs.658889**	**0.4229**
**IGF2BP3**	**insulin-like growth factor 2 mRNA binding protein 3**	**Hs.700696**	**0.3822**
TACC1	transforming, acidic coiled-coil containing protein 1	Hs.279245	0.3138
SMAD5	SMAD family member 5	Hs.167700	0.3442
PPIG	peptidylprolyl isomerase G (cyclophilin G)	Hs.727580	0.3724
NPHP3	nephronophthisis 3 (adolescent)	Hs.511991	0.3404
MYCBP2	MYC binding protein 2, E3 ubiquitin protein ligase	Hs.591221	0.437
MKI67	antigen identified by monoclonal antibody Ki-67	Hs.80976	0.3288
FBXO9	F-box protein 9	Hs.216653	0.3038
ERBB3	v-erb-b2 erythroblastic leukemia viral oncogene homolog 3 (avian)	Hs.118681	0.4789
BRIP1	BRCA1 interacting protein C-terminal helicase 1	Hs.128903	0.3294
ATF6	activating transcription factor 6	Hs.492740	0.4689
BRCC3	BRCA1/BRCA2-containing complex, subunit 3	Hs.558537	0.4801

Supplementary Table S2: Antibodies used in this study

Antibody	Clone & host	Dilution	Company
CD133	W6B3C1, mouse IgG1	1:50 for IF	MACS
CD133	W6B3C1, mouse IgG1	1:100 for WB	MACS
ABCG2	SC-58222, mouse IgG2a	1:50 for IF	Santa Cruz
ABCG2	SC-58222, mouse IgG2a	1:200 for WB	Santa Cruz
ABCB1	SC-13131, mouse IgG2b	1:50 for IF	Santa Cruz
ABCB1	SC-13131, mouse IgG2b	1:100 for WB	Santa Cruz
IGF2BP3	HPA002037, rabbit IgG	1:100 for IF	Sigma-Aldrich
IGF2BP3	HPA002037, rabbit IgG	1:500 for WB	Sigma-Aldrich
HRP-β-actin	AC-15, mouse	1:20,000 for WB	Sigma-Aldrich
HRP-anti mouse IgG1	Goat	1:3,000 for WB	Santa Cruz
HRP-anti mouse IgG2a	Goat	1:3,000 for WB	Santa Cruz
HRP-anti Rabbit IgG	Goat	1:4,000 for WB	Sigma-Aldrich
HRP-anti mouse IgG2b	Goat	1:3,000 for WB	Santa Cruz

WB: Western blotting; IF: Immunofluorescence

Supplementary Table S3: Primers used for Real-time PCR and other sequences

Gene	GenBank no.	Forward (5′-3′)	Reverse (5′-3′)
CD133	NM_001145	tggatgcagaacttgacaacgt	atacctgctacgacagtcgtggt
Nanog	NM_024865	aatacctcagcctccagcagatg	tgcgtcacaccattgctattcttc
Oct4	NM_002701	cttgctgcagaagtgggtggaggaa	ctgcagtgtgggtttcgggca
LGR5	NM_001277	aacagtcctgtgactcaactcaag	ttagagacatgggacaaatgccac
ABCG2	NM_004827	tcatcagcctcgatattccatct	ggcccgtggaacataagtctt
ABCB1	NM_000927	gctcctgactatgccaaagc	tcttcacctccaggctcagt
IGF2BP3	NM_006547	actgcacgggaaacccatag	tgtctagtgcttgtctagcttgg
IGF2BP1	NM_006546	ggcctgagaatgagtg	gaggggcagacagtgttg
GAPDH	NM_002046	agaaggctggggctcatttg	aggggccatccacagtcttc
